# Cigarette Smoke Alters the Hematopoietic Stem Cell Niche

**DOI:** 10.3390/medsci2010037

**Published:** 2014-02-18

**Authors:** Robert W. Siggins, Fokhrul Hossain, Tayyab Rehman, John N. Melvan, Ping Zhang, David A. Welsh

**Affiliations:** 1 Department of Physiology, Louisiana State University Health Sciences Center, New Orleans, LA 70112, USA; 2 Comprehensive Alcohol Research Center, Louisiana State University Health Sciences Center, New Orleans, LA 70112, USA; 3 Alcohol and Drug Abuse Center of Excellence, Louisiana State University Health Sciences Center, New Orleans, LA 70112, USA; 4 Department of Biochemistry and Molecular Biology, Louisiana State University Health Sciences Center, New Orleans, LA 70112, USA; 5 Department of Medicine, Section of Pulmonary and Critical Care Medicine, Louisiana State University Health Sciences Center, New Orleans, LA 70112, USA; 6 Department of Surgery, Michigan State University, East Lansing, MI 48824, USA

**Keywords:** cigarette smoke, hematopoiesis, niche, mesenchymal stromal cell, hematopoietic stem cell

## Abstract

Effects of tobacco smoke on hematologic derangements have received little attention. This study employed a mouse model of cigarette smoke exposure to explore the effects on bone marrow niche function. While lung cancer is the most widely studied consequence of tobacco smoke exposure, other malignancies, including leukemia, are associated with tobacco smoke exposure. Animals received cigarette smoke exposure for 6 h/day, 5 days/week for 9 months. Results reveal that the hematopoietic stem and progenitor cell (HSPC) pool size is reduced by cigarette smoke exposure. We next examined the effect of cigarette smoke exposure on one supporting cell type of the niche, the mesenchymal stromal cells (MSCs). Smoke exposure decreased the number of MSCs. Transplantation of naïve HSPCs into irradiated mice with cigarette smoke exposure yielded fewer numbers of engrafted HSPCs. This result suggests that smoke-exposed mice possess dysfunctional niches, resulting in abnormal hematopoiesis. Co-culture experiments using MSCs isolated from control or cigarette smoke-exposed mice with naïve HSPCs *in vitro* showed that MSCs from cigarette smoke-exposed mice generated marked expansion of naïve HSPCs. These data show that cigarette smoke exposure decreases *in vivo* MSC and HSC number and also increases pro-proliferative gene expression by cigarette smoke-exposed MSCs, which may stimulate HSPC expansion. These results of this investigation are clinically relevant to both bone marrow donors with a history of smoking and bone marrow transplant (BMT) recipients with a history of smoking.

## 1. Introduction

While pulmonary disorders are the most obvious manifestation of cigarette smoke-induced disease, there is a growing appreciation that smoking and chronic lung disease are associated with significant systemic consequences and comorbidities. Distal organ and tissue injury, linked to systemic inflammation and oxidative stress, commonly result in skeletal myopathy, cardiovascular disease, osteoporosis, depression and metabolic derangement.

Stem and progenitor cells play a pivotal role in tissue maintenance and repair in response to injury, and it has been proposed that resident and/or hematopoietic progenitor cell perturbations may be pathogenic in smoking-related emphysema [1]. Further, circulating endothelial and hematopoietic progenitors are particularly reduced in chronic obstructive pulmonary disease patients with low body mass index, suggesting that systemic disease is related to stem cell function [2].

Hematologic sequelae of tobacco smoke exposure include CD8^+^ cytotoxic T cell lymphocytosis and neutrophilia. BrdU labeling studies show decreased transit time through the post-mitotic pool of the bone marrow, consistent with chronic stimulation of hematopoiesis [3]. Despite the egress of maturing cells from the bone marrow, several studies have found fewer circulating CD34^+^ progenitor/stem cells in both smokers and patients with chronic lung disease [2,4–6]. Limited and conflicting data, using colony forming assays for hematopoietic progenitors, are available describing the effects of cigarette smoke and its constituents on bone marrow stem cells [7,8].

Within the bone marrow, hematopoiesis occurs within unique niches composed of many cell types including mesenchymal stromal cells (MSCs) and their progeny, endothelial cells, as well as the extracellular milieu [9]. Through the production of adhesion and soluble factors, MSCs participate in the regulation of activation and function of both long-term repopulating hematopoietic stem cells (LT-HSCs) and hematopoietic progenitor cells, which includes the activated short-term repopulating hematopoietic stem cells (ST-HSCs). LT-HSCs are capable of repopulating the immune system through serial bone marrow transplantations, while ST-HSCs have limited repopulation capacity [10]. The use of signaling lymphocyte activation molecule (SLAM) family of cell surface proteins has been shown to highly enrich LT-HSCs in mice studies [11]. Previous studies have shown that MSCs and their progeny express the alpha7 nicotinic receptor subunit and respond to nicotine, the primary active ingredient in cigarettes, in a dose-dependent manner [12–14]. Additionally, nicotine has been shown to perturb pre- and post-natal hematopoiesis [15]. *In vitro* low dose nicotine leads to enhanced survival, whereas high doses impair MSC differentiation and enhance apoptosis [7]. Exposure to unfractionated cigarette smoke extract also decreases both MSC differentiation and survival. Chronic smoke exposure’s impact on MSC-HSPC communication and subsequent effects on HSPC status remain unknown. We hypothesized that chronic smoke exposure decreases the bone marrow HSPC pool by perturbing the stem cell niche in a murine model. The results of this study may impact both bone marrow donors and BMT recipients.

## 2. Experimental Section

### 2.1. Animals

Female C57BL/6 mice (8 weeks old; National Cancer Institute) were housed (5/cage) in a specific pathogen-free facility with a 12 h light/dark cycle. Mice were maintained on a standard laboratory diet. Enhanced GFP (eGFP) transgenic mice (C57BL/6-Tg(UBC-GFP)30Scha/J; The Jackson Laboratory, Bar Harbor, ME, USA) were bred in the Louisiana State University Health Sciences Center, New Orleans animal care facility following an approved Institutional Animal Care and Use Committee (IACUC) protocol. The LSUHSC IACUC approved all experimental procedures in adherence to the National Institutes of Health guidelines prior to the initiation of experiments.

### 2.2. Cigarette Smoke Exposure

Smoke-exposed mice (mean ± SE: pre-exposure body weight 18.21 ± 0.16 g; post-exposure weight 25.37 ± 0.40 g; *n* = 20) were transferred to separate smoking cages and placed into Plexiglas^®^ chambers (model TE-10, Teague Enterprises, Davis, CA, USA). Animals were next exposed to whole body mainstream and side stream smoke via inhalation from 3R4F research cigarettes (University of Kentucky, Lexington, KY, USA) at 100 mg/m^3^ total suspended particles (TSP) for 6 h/day, 5 days/week for 9 months. Control mice (pre-exposure body weight 18.24 ± 0.20 g; post-exposure weight 28.33 ± 1.17; *n* = 10) were kept in identical cages and exposed to filtered air in parallel with the experimental group.

### 2.3. Hematopoietic Bone Marrow Cell Isolation

Following 9 months of smoke exposure, femurs were isolated from both control and smoke exposure groups. Hematopoietic bone marrow cells (BMCs) were collected by centrifugation at 500× *g* for 5 min. Femurs were then flushed with 1 mL of PBS to collect any remaining cells. BMCs were next filtered through a 70 μm nylon cell strainer (BD Biosciences, San Jose, CA, USA). Erythrocytes were lysed with 1 mL RBC Lysis Solution (Qiagen, Valencia, CA, USA) for 3 min, washed with 3 mL PBS and centrifuged at 500× *g* for 5 min. Cell pellets were decanted and resuspended in 1 mL PBS for quantification and viability assessment with a hemocytometer by trypan blue exclusion.

### 2.4. Mesenchymal Stromal Cell Isolation and Colony Forming Unit-Fibroblast (CFU-f) Assays

Mesenchymal stromal cells (MSCs) were isolated from the bone marrow of 9 months smoke-exposed or control mice using two different methods. Briefly, bones (both femur and tibia) were crushed with pestle and digested with 0.25% collagenase 1 (Collagenase type 1, 290 U/mg, Worthington, Lakewood, NJ, USA). After digestion, cells were filtered through a 70-μm cell strainer (BD Biosciences), centrifuged at 500× *g* for 5 min, and resuspended in Complete Mesencult^®^ Medium (StemCell Technologies, Vancouver, Canada). For the second method, bone marrow cells were collected by centrifugation of both femur and tibia at 500× *g* for 5 min. The bones were then flushed with 1 mL of sterile PBS to collect any remaining cells. Then cells were filtered through a 70-μm cell strainer and resuspended as stated above. Five million cells (in 2 mL media/well) were then plated in 6 well plates and incubated in a hypoxia incubator (5% O_2_ with 5% CO_2_) at 37 °C. MSCs are highly sensitive to atmospheric oxygen and have been shown to have enhanced proliferation when cultured with lower oxygen concentration. Upon achieving 70%–80% confluency, cells were released with 0.25% trypsin and aliquots preserved in freeze media (90% heat inactivated FBS + 10% DMSO) and stored in liquid N_2_ for future use. CFU-f assays were performed by incubating 10^6^ cells (2 mL media/well) in 6 well plates under hypoxic conditions (5% CO_2_) at 37 °C. After two weeks of incubation, cells were Giemsa stained and colony counts determined. All experiments utilized the crush method for harvesting MSCs.

### 2.5. Cell Immunostaining and Flow Cytometry

BMCs were analyzed for hematopoietic stem and progenitor cell (HSPC) and MSC phenotypes using multicolor flow cytometry. HSPCs were defined by lineage (Lin−/stem cell factor receptor (ckit)+/stem cell antigen-1 (Sca-1)+ and, long-term repopulating hematopoietic stem cells (LT-HSCs) were defined using signaling lymphocyte activation molecule (SLAM) family (Lin-CD48-CD150+) [11]. Nucleated BMCs were stained with monoclonal antibodies in StemSpan Serum-free Media (1 × 10^6^ cells in 100 μL). All antibodies were purchased from eBioscience Inc. (San Diego, CA, USA) unless otherwise stated. Mouse biotin-conjugated hematopoietic lineage panel (10 μg/mL each) was added to BMCs and included CD3 (clone:145-2c11), CD45R/B220 (clone RA3-6B2), CD11b (clone M1/70), TER-119 (cloneTER-119), and Ly-6G (clone RB6-8c5) (eBioscience) or isotype control antibodies. Cells were incubated at room temperature (RT) in the dark for 15 min. Cells were washed in 2 mL of PBS at 500× *g* for 5 min, decanted, and resuspended in a mixture of PE-TxR-streptavidin (10 μg/mL; BD Biosciences) and fluorochrome-conjugated anti-mouse ckit (APC-AF750; clone 2B8), Sca-1 (PE-Cy5.5; clone D7), SLAM markers CD48 (APC; clone HM 48-1) and CD150 (PE; clone mshad 150), or isotype matched controls. BMCs were again incubated at RT in the dark for an additional 15 min. Cells were washed and resuspended in a final volume of 250 μL of PBS + 1% paraformaldehyde (PFA). Analysis was performed on a LSR-II flow cytometer with FACSDiva^™^ software [16]. All quantification was done on live cells only (LIVE/DEAD Fixable Dead Cell Stain, Life Technologies, Grand Island, NY, USA).

### 2.6. Bone Marrow Repopulation Assay

To measure HSPC repopulating ability, 9 months smoke-exposed and control mice were irradiated with 8 Gy (Mark 1 Irradiator, JL Shepherd & Associates, San Fernando, CA, USA) at 425 Rad/min for 1.88 min. Four hours after radiation, animals were transplanted with eGFP bone marrow cells (10^6^) from 7–8 weeks old GFP transgenic mice (no smoke exposure) via jugular vein injection. Animals were not exposed to smoke after transplantation. Twenty weeks after transplantation, mice were sacrificed to collect bone marrow cells according to the protocol *vida supra*. GFP positive bone marrow cells were analyzed for HSPC engraftment by flow cytometry as aforementioned.

### 2.7. Mesenchymal and Hematopoietic Progenitor Cell Co-Culture

Passage 1 MSCs isolated from control and smoke-exposed mice were thawed and seeded onto 24 well plates in 1 mL of media at 25,000 cells/mL and incubated for 24 h in a humidified incubator at 37 °C and 5% CO_2_. Lineage negative cells were isolated from 7–8 weeks old eGFP transgenic mice (female) using a lineage cell depletion kit (Miltenyi Biotec Inc, Auburn, CA, USA) according to the manufacturer’s protocol. Lin-cells (10^4^) were co-cultured with MSCs in StemSpan SFEM media (Stemcell Technologies, Vancouver, Canada) + 2% HI FBS. Cells were harvested and then analyzed using an LSRII flow cytometer (BD Biosciences) on day 1, 4, and 8 of co-culture. Only GFP+ cells were analyzed for HSPC populations.

### 2.8. Quantitative mRNA Expression

MSCs were co-cultured as described above along with two additional groups of control and smoke-exposed MSCs without eGFP+ HSPCs. After 24 h of co-culture, wells were washed with 0.5 mL of Cell Dissociation Solution (non-enzymatic, 1×; Sigma) for 1 min to remove HSPCs. Removal of eGFP+ cells was confirmed by fluorescence microscopy. Persistently adherent cells were then washed twice with PBS followed by trypsinization. The cells were then lysed and reverse transcription (RT) performed using the TaqMan^®^ Gene Expression Cells-to-C_T_^™^ Kit (Ambion, Grand Island, NY, USA) according to the manufacturer’s protocol with one modification: RQ1 RNase-free DNase (Promega, Madison, WI, USA) was used in place of Ambion DNase. Real-time PCR was performed using 5 μL of a 1:5 dilution of either RT or no RT (NRT) reactions with RT^2^ SYBR^®^ Green qPCR Mastermix (Qiagen, Valencia, CA, USA) according to the manufacturer’s protocol. Gene expression (gene primers; Table 1) was measured using the iCycler iQ^™^ Real-time PCR Detection System (Bio-Rad, Hercules, CA, USA) and the 2^−ΔΔCT^ method was used to determine fold change with 18S rRNA as the housekeeping control. All primers were designed to cross exon-exon junctions to avoid possible amplification of residual DNA.

### 2.9. Statistical Analysis

Data are presented as mean ± SEM. The sample size is indicated in each figure legend. All statistical analysis was performed using GraphPad Prism Software^™^ [17]. Statistical analysis was conducted using unpaired Student’s *t*-test, non-parametric Mann-Whitney *t*-test, or two-way ANOVA, followed by Bonferroni post-tests (for comparisons between multiple groups). Asterisks and different letters denote statistical significance with *p* < 0.05.

## 3. Results and Discussion

The current study examined the effects of cigarette smoke exposure on hematopoietic stem and progenitor cells (HSPCs) and on the hematopoietic stem cell niche in the bone marrow. We found that smoke exposure decreased HSPCs and showed a trend for decreased long-term repopulating hematopoietic stem cells (LT-HSCs), as defined by lineage (Lin)-c-kit+Sca-1+ (LKS) and signaling lymphocyte activation molecule (SLAM; Lin-CD48-CD150+) marker phenotypes, respectively [10,11]. This observed decrease in frequency of both ST-HSCs and LT-HSCs could be clinically significant for BMTs when using bone marrow donated from patients with a history of cigarette smoke exposure. Moreover, MSCs, the prototypic niche cell, were diminished in animals exposed to smoke and, as evidenced by repopulation studies, the smoke-exposed bone marrow is ill equipped to support HSPCs. These findings are further supported by *ex vivo* studies showing that key MSC-produced molecules are perturbed by smoke exposure and that smoke-exposed MSCs accelerate HSPC proliferation, providing an explanatory mechanism for the diminished bone marrow HSPC counts.

The niche comprises mesenchymal stromal cells (MSCs) and their progeny, endothelial cells, extracellular matrix, and soluble mediators [18]. The niche MSC lineage plays a major role in instructing HSPC fate, through either quiescence and self-renewal or lineage commitment toward mature blood cell types. Effects of cigarette smoke on the structure and function of the niche have yet to be explored.

Published evidence of the effects of cigarette smoke on various bone marrow cell populations have shown mixed results. Data from these studies demonstrated opposing effects of cigarette smoke extract and nicotine alone on function of MSCs and their progeny [7,19–21]. Other studies have reported dose-dependent effects of nicotine alone, with low dose nicotine enhancing MSC activity and osteoblastogenesis, while high dose nicotine inhibits these functions [12–14]. To date, only a handful of publications have assessed *in vivo* effects of smoke exposure on the hematopoietic microenvironment [8,22,23]. These studies have consistently shown enhanced hematopoietic activity as assessed by accelerated neutrophil differentiation and transit into the circulation [24]. Additionally, nicotine has been shown to lead to enhanced extramedullary hematopoiesis, which suggests disruption of the physiological functioning of the bone marrow niche microenvironment [8].

### 3.1. Cigarette Smoke Exposure Decreases the Number of Bone Marrow HSPCs

**Figure 1:**
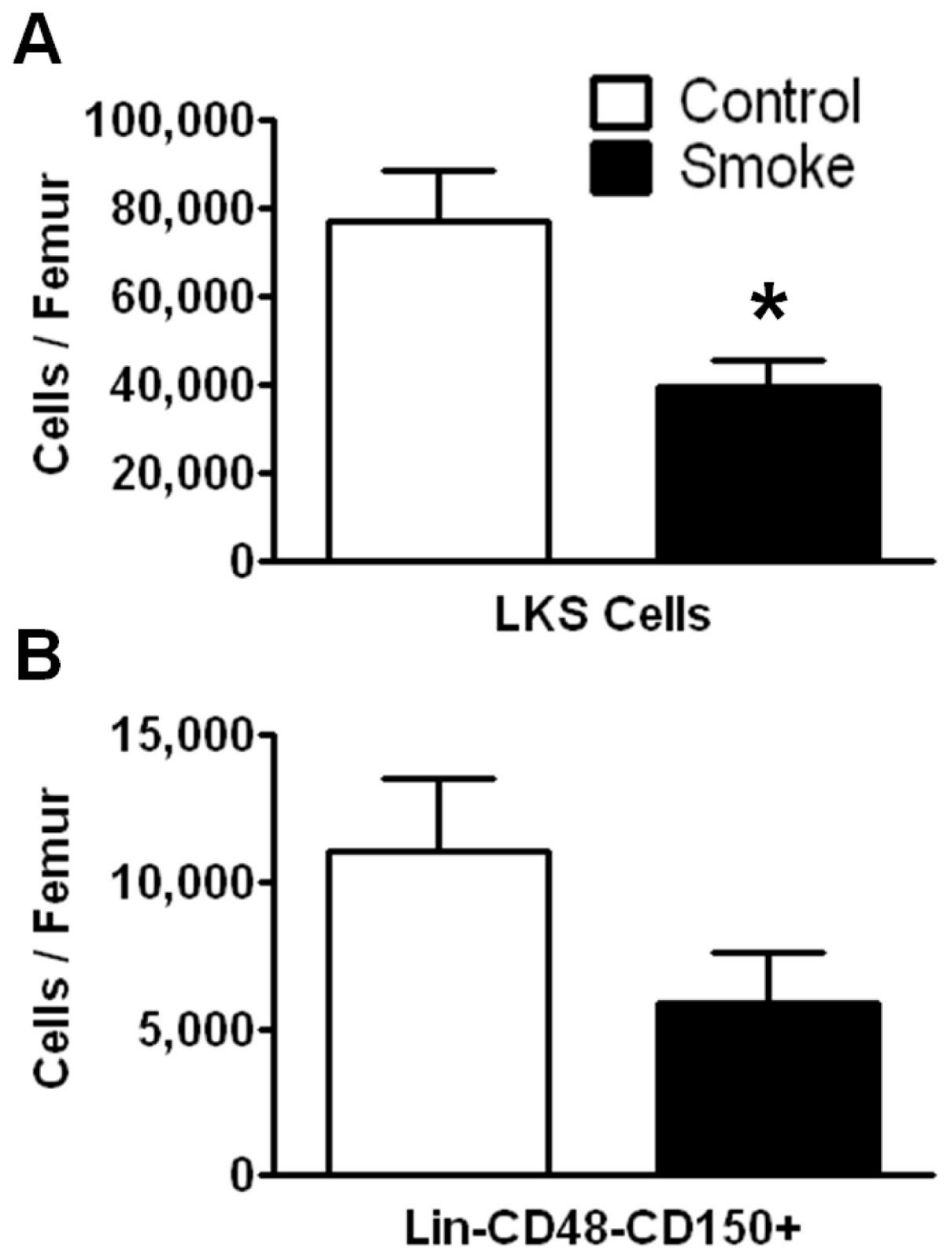
Flow cytometric analysis of hematopoietic stem/progenitor cells (HSPCs) following 9 months of control or smoke exposure. (**A**) Total Lin-c-kit+Sca-1+ (LKS; short-term repopulating hematopoietic stem cells (ST-HSCs)) per femur of control and smoke exposed mice; (**B**) Long-term (LT)-HSC identified by signaling lymphocyte activation molecule (SLAM) markers (Lin-CD48-CD150+) support the trend observed in the ST-HSC population. * *p* < 0.05; *N* = 12 for each group.

To investigate the effects of long term (9 months) smoke exposure on the quantity of bone marrow HSPCs, BMCs were analyzed by flow cytometry based on their cell surface marker expression. Bone marrow lineage negative (Lin^−^) c-kit^+^Sca-1^+^ cells are enriched for hematopoietic stem cells, however the majority of these cells are short-term repopulating stem cells (ST-HSCs) and hematopoietic progenitor cells [25]. Our results show that Lin^−^c-kit^+^Sca-1^+^ cells were significantly fewer in the smoke-exposed bone marrow compared to controls (Figure 1A). We found no difference in the total number of BMCs between groups.

The SLAM family of cell surface markers (CD150 and CD48) has been shown to specifically identify murine long-term hematopoietic stem cells (LT-HSCs) [26]. This phenotype is maintained even under stress conditions in mice [11,27]. Employing SLAM markers, we found a trend for a decrease in total LT-HSCs in the cigarette smoke-exposed animals, but this data failed to reach significance (*p* = 0.1124; Figure 1B).

#### 3.1.1. Cigarette Smoke Decreases the Number of Bone Marrow MSCs

**Figure 2:**
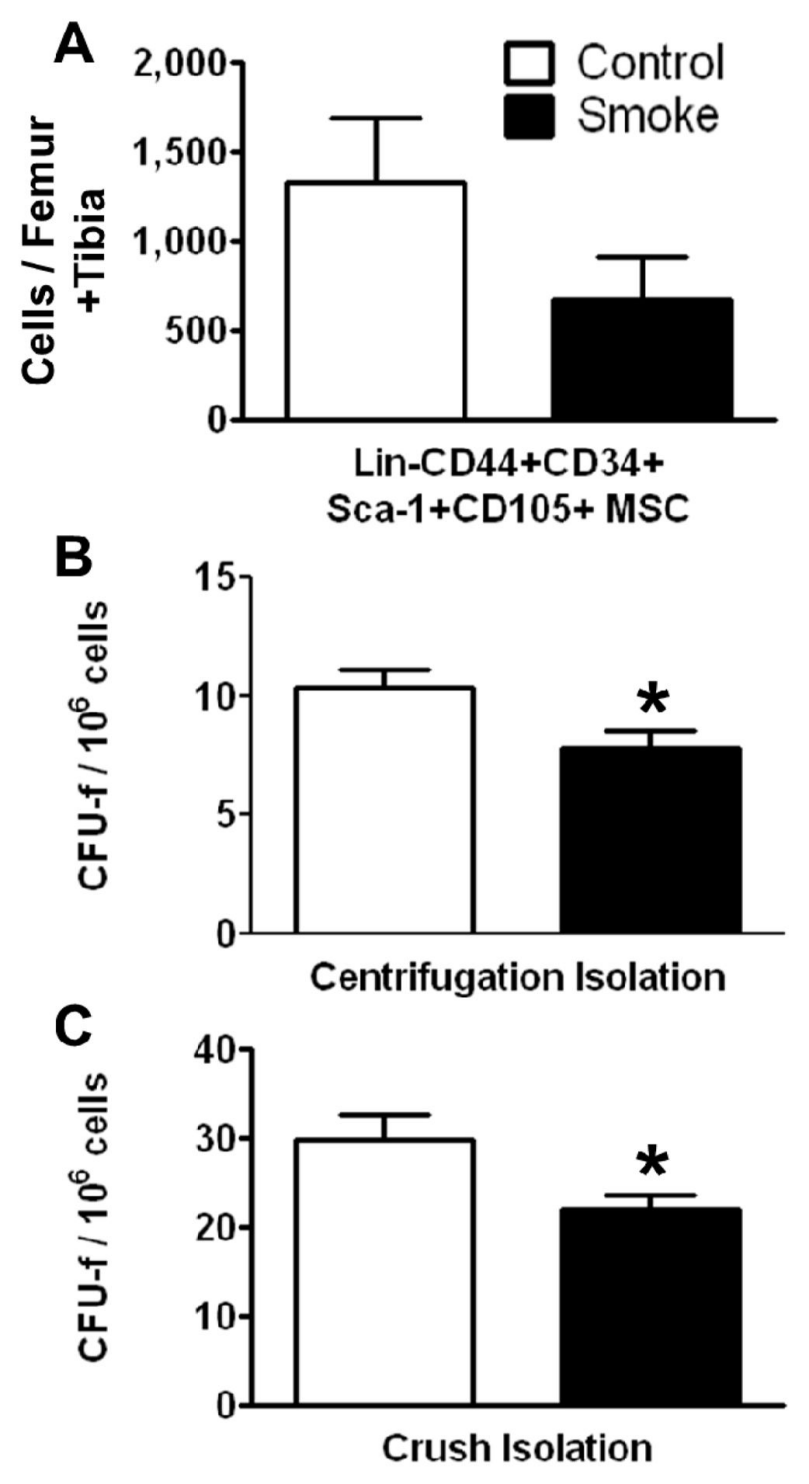
Smoke exposure decreases bone marrow mesenchymal stromal cells (MSCs). (**A**) Fluorescence-activated cell sorting (FACS) analysis of MSCs by surface phenotype; (**B**, **C**) Functional enumeration, based on colony forming unit-fibroblast (CFU-f) assays, is decreased in MSCs from smoke exposed animals. * *p* < 0.05; *N* = 6 for both groups.

Effects of cigarette smoke exposure on MSC number was assessed by FACS analysis and colony forming unit-fibroblast (CFU-f) activity were determined using the MesenCult^®^ culture system. FACS analysis of MSCs isolated using the crush method showed a trend for decreased numbers of total Lin-CD44+CD34+Sca-1+CD105+ cells. Both the marrow centrifugation with flushing method and the compact bone crush method were performed to isolate MSCs. Compared with control animals, smoke-exposed mice had significantly fewer CFU-f following both isolation methods (Figure 2B, C). However, we found more CFU-f using the compact bone crush method than centrifugation plus flushing, as expected. These results suggest that smoking reduces either the frequency of MSCs, the ability of MSCs to form colonies, or both. As the compact bone crush method isolates greater numbers of MSCs from bone, we utilized this isolation method for the remainder of the studies.

#### 3.1.2. Cigarette Smoke Impairs the Engraftment of HSPCs in the Bone Marrow

**Figure 3:**
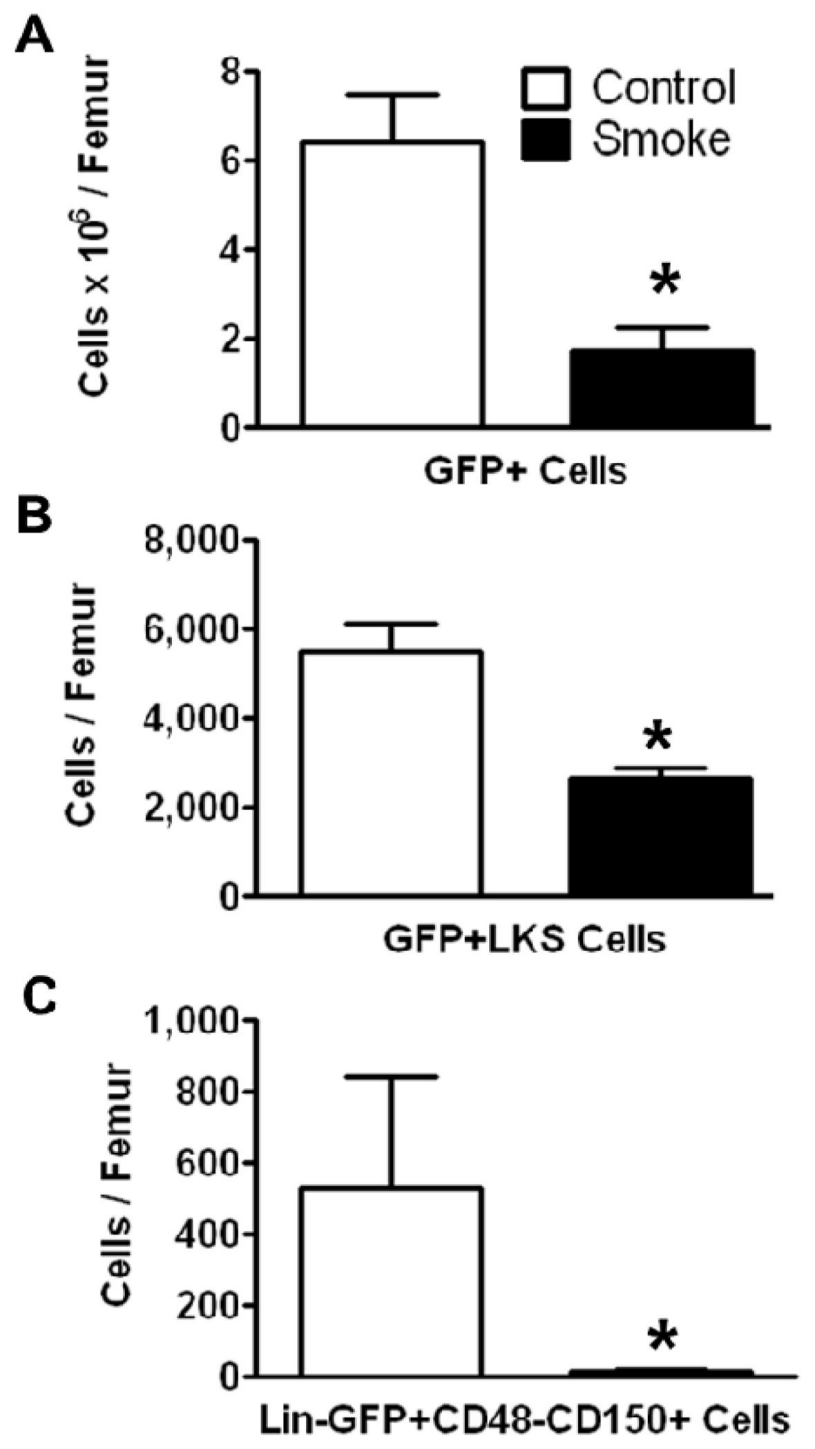
Smoke exposure decreases cell engraftment after bone marrow transplantation. (**A**) Total GFP+ bone marrow cells; (**B**) GFP+ ST-HSCs; and (**C**) GFP+ LT-HSCs from control and cigarette smoke-exposed animals 20 weeks post-GFP+ bone marrow transplant. * *p* < 0.05; *N* = 6 for both groups.

To investigate the effects of smoking on HSPC engraftment, we reconstituted irradiated mice (both smoke-exposed and age-matched controls) with bone marrow cells from C57BL/6 eGFP-transgenic mice. The animals were not re-exposed to cigarette smoke after transplantation. Twenty weeks post-transplantation, HSPCs were analyzed by flow cytometry. The number of engrafted eGFP+ cells in the bone marrow of smoke-exposed mice was significantly reduced as compared to control animals (Figure 3A). GFP+ ST-HSCs were significantly greater in controls animals than in smoke-exposed mice (Figure 3B). Additionally, the GFP+ LT-HSCs were significantly reduced in the cigarette smoke-exposed bone marrow (Figure 3C).

#### 3.1.3. Smoke-Exposed MSCs Promote Expansion of Primitive Bone Marrow Cells

**Figure 4:**
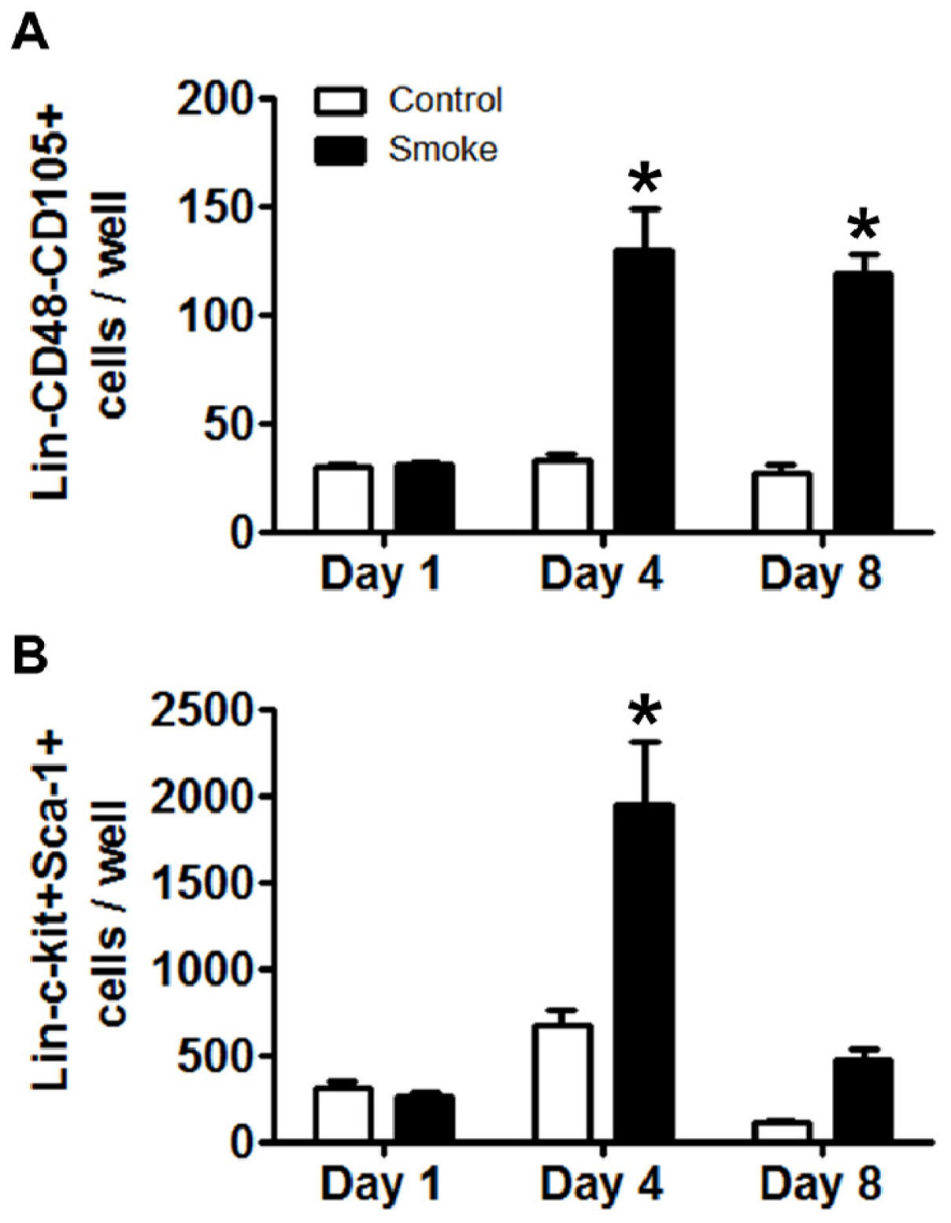
Co-culture of Lin-GFP+ HSPCs with MSCs isolated from control and smoke-exposed animals. (**A**) MSCs isolated from smoke-exposed animals leads to increased numbers of Lin-GFP+CD48-CD150+ cells following 4 and 8 days of co-culture; (**B**) Lin-c-kit+Sca-1+ ST-HSCs are increased after 4 days of co-culture in the smoke-exposed MSC group, but return to day 1 levels after 8 days. * *p* < 0.05; *N* = 5 for each group.

Recent publications suggest that human MSCs support *ex vivo* expansion of HSPCs [28–30]. As an *ex vivo* model of a simplified hematopoietic stem cell niche, Lin-bone marrow cells from healthy, eGFP+ transgenic mice were co-cultured on monolayers of MSCs from control and smoke-exposed mice. eGFP+ HSPCs were assessed serially by flow cytometry. The total number of eGFP+Lin-CD48-CD150+ cells was significantly increased at day 4 and day 8 in wells containing smoke-exposed MSCs (Figure 4A). Co-culture with MSCs from smoke-exposed animals induced an expansion of eGFP+Lin-c-kit+Sca-1+ cells at day 4 but this cell type returned to baseline at day 8 (Figure 4B). One function of MSCs is to participate in maintaining HSC self-renewal [31,32]. These results demonstrate that smoke-exposed MSCs induced a pronounced expansion of Lin-CD48-CD150+ cells, which may be related to impaired HSPC differentiation. This hypothesis warrants further examination.

#### 3.1.4. Cigarette Smoke Perturbs MSC Expression of Niche Molecules

MSCs play a pivotal role in maintaining homeostasis of the bone marrow niche through the expression of an array of signaling molecules. As MSC niche molecule expression is informed by feedback interaction with target cells, experimental and control MSCs were co-cultured for 24 h with eGFP+Lin− bone marrow cells from healthy mice. Removal of eGFP+ hematopoietic cells was confirmed by inspection for residual fluorescence after washing and, total MSC RNA was extracted to determine their gene expression of putative regulators of niche function (Table 2) [18,33]. We found the highest expression of Angiopoietin 1 (Angpt1) in control MSCs co-cultured with HSPCs. Smoke-exposed MSCs with or without co-culture showed a 60% reduction in gene expression as compared to control. Bone morphogenic protein 4 (BMP4) and Cxcl12 were both decreased in smoke-exposed MSC groups. MSCs from smoke-exposed mice had decreased expression of Dikkopf 2 (Dkk2) and showed over a 200% increase in Jagged 1 (Jag1) expression. Finally, platelet-derived growth factor alpha (Pdgfa) showed a 143% increase in expression from MSCs isolated from smoke-exposed mice. These data indicate that smoke-exposed MSCs have aberrant gene expression across multiple pathways that are known to regulate hematopoiesis, and likely contribute to a dysfunctional niche.

## 4. Conclusions

These data support our hypothesis that hematologic changes in cigarette smokers are caused by damage to niche cells, specifically MSCs and their progeny. We have shown that smoke exposure decreased HSPC pool size and concomitantly deceased MSC number. The decreased number of MSCs corresponded to a decrease in naïve HSPC engraftment after bone marrow transplantation in smoke-exposed animals. In spite of this decrease in engraftment and number of HSPCs and MSCs, smoke exposure also altered MSC gene expression of pro-proliferative signaling proteins. Our data thus suggest that smoke exposure leads to dysregulation of hematopoiesis through disruption of MSC signaling pathways in addition to decreasing MSC number. This may serve as one mechanism by which smoke exposure promotes greater peripheral leukocyte production in cigarette smokers. These data also offer potential targets of therapeutic intervention with regards to both bone marrow donor and transplant recipient with a history of cigarette smoke exposure.

## Figures and Tables

**Table 1:** Primer sequences used for reverse transcription (RT)-qPCR analysis of niche mediators. 18S rRNA was used as a loading control to normalize results. Abbreviations: Angiopoietin 1 (Angpt1); Bone morphogenic protein 4 (Bmp4); *N*-cadherin (Cdh2); Chemokine (C-X-C motif) ligand 12 (Cxcl12); Dikkopf 2 (Dkk2); Jagged 1 (Jag1); Platelet-derived growth factor alpha (Pdgfa).

Gene Symbol	Forward Primer	Reverse Primer
**18S**	ATTCGAACGTCTGCCCTATCA	GTCACCCGTGGTCACCATG
**Angpt1**	GAGGATTGAGCTGATGGACTG	ACCGTGTAAGATCAAGCTGC
**Bmp4**	GAGCAGAGCCAGGGAAC	GAAGAGGAAACGAAAAGCAGAG
**Cdh2**	GGACAGCCCCTTCTCAATG	TTCTCACAGCATACACCGTG
**Cxcl12**	CGCTCTGCATCAGTGACG	TGAAGGGCACAGTTTGGAG
**Dkk2**	CAGTCAGCCAACCGATCTG	CTTCCAACTTCACATTCCTTATCAC
**Jag1**	CGAACCCCTGTCATAATGGAG	ACAGGTCCCGCTATTGTAAC
**Pdgfa**	GACCTCCAGCGACTCTTG	CCTCAATACTTCTCTTCCTGCG

**Table 2:** RT-qPCR analysis of MSC niche mediators from control and smoke-exposed animals were cultured alone or co-cultured with naïve Lin-GFP+ HSPCs for 1 day. Smoke exposure led to down regulation of all but two of the analyzed genes, Jagged1 and Platelet-derived growth factor-α. Data are expressed as a percent change from control ± SEM (MSC alone). Different letters in parenthesis next to the percent values signify statistical significance; *p* < 0.05. Abbreviations: Angiopoietin 1 (Angpt1); Bone morphogenic protein 4 (Bmp4); *N*-cadherin (Cdh2); Chemokine (C-X-C motif) ligand 12 (Cxcl12); Dikkopf 2 (Dkk2); Jagged 1(Jag1); Platelet-derived growth factor alpha (Pdgfa).

Gene Symbol	MSC	Co-Culture	MSC-CSE	Co-Culture-CSE
**Angpt1**	100 ± 18 (a)	356 ± 110 (b)	40 ± 8 (a)	41 ± 12 (a)
**Bmp4**	100 ± 44 (a)	113 ± 30 (a)	18 ± 6 (a)	34 ± 21 (a)
**Cdh2**	100 ± 9 (a,b)	110 ± 23 (b)	64 ± 5 (a,b)	40 ± 13 (a)
**Cxcl12**	100 ± 12 (a)	51 ± 13 (b,c)	69 ± 7 (a,b)	18 ± 7 (c)
**Dkk2**	100 ± 4 (a)	50 ± 20 (b)	26 ± 8 (b,c)	9 ± 3 (c)
**Jag1**	100 ± 12 (a)	31 ± 5 (a)	203 ± 37 (b)	26 ± 9 (a)
**Pdgfa**	100 ± 3 (a)	43 ± 5 (b)	143 ± 18 (c)	29 ± 9 (b)
